# Highly defended nudibranchs “escape” to visually distinct background habitats

**DOI:** 10.1093/beheco/arae053

**Published:** 2024-07-04

**Authors:** Cedric P van den Berg, Matteo Santon, John A Endler, Karen L Cheney

**Affiliations:** School of the Environment, The University of Queensland, University Drive, St. Lucia, QLD 4072, Australia; School of Biological Sciences, University of Bristol, 24 Tyndall Ave, Bristol, BS8 1TQ, United Kingdom; School of Biological Sciences, University of Bristol, 24 Tyndall Ave, Bristol, BS8 1TQ, United Kingdom; School of Life and Environmental Sciences, Deakin University, Waurn Ponds, VIC 3216, Australia; School of the Environment, The University of Queensland, University Drive, St. Lucia, QLD 4072, Australia

**Keywords:** aposematism, camouflage, defensive animal coloration, escape and radiate hypothesis, signal honesty, visual ecology, visual modeling, warning signals

## Abstract

The “escape and radiate” hypothesis predicts that once species have evolved aposematism, defended species can utilize more visually diverse visual backgrounds as they “escape” the need to be well camouflaged. This enables species to explore new ecological niches, resulting in increased diversification rates. To test this hypothesis “escape” component, we examined whether the background habitats of 12 nudibranch mollusk species differed among species depending on the presence and strength of chemical defenses. We obtained a rich array of color pattern statistics using quantitative color pattern analysis to analyze backgrounds viewed through the eyes of a potential predator (triggerfish, *Rhinecanthus aculeatus*). Color pattern analysis was done at viewing distances simulating an escalating predation sequence. We identified 4 latent factors comprising 17 noncorrelated color pattern parameters, which captured the among-species variability associated with differences in chemical defenses. We found that chemically defended species, indeed, were found on visually distinct backgrounds with increased color and luminance contrast, independent of viewing distance. However, we found no evidence for increased among-species background diversity coinciding with the presence and strength of chemical defenses. Our results agree with the “escape and radiate” hypothesis, suggesting that potent chemical defenses in Dorid nudibranchs coincide with spatiochromatic differences of visual background habitats perceived by potential predators.

## Introduction

Animals and plants use aposematic color patterns to advertise defenses to potential predators (Poulton 1890). One of the hypothesized benefits of an organism evolving aposematism is the potential to “escape” the costs of crypsis, including limited movement and thus access to resources due to the need to match a specific background habitat or preventing detection through motion contrast for primary defense (Endler 1984; Regan and Beverley 1984; Merilaita and Tullberg 2005). Therefore, Merilaita and Tullberg (2005) suggested that alternative visual defense strategies, such as aposematism, are more likely to evolve in highly variable visual environments where crypsis through background matching is difficult to achieve. The evolution of aposematic signaling provides increased access to resources across ecological niches as individuals are not strongly restricted to particular habitats to avoid predator detection. This reduced dependency on crypsis may drive increased speciation and diversification rates (Ehrlich and Raven 1964; Bulmer 1972; Arbuckle and Speed 2015). This process was coined “escape and radiate” by Thompson (1989) and has since been adopted as a core concept underpinning the role of chemical defenses in adaptive radiations (e.g. Arbuckle and Harris 2021).

The evolution of potent chemical defenses often coincides with aposematism (see Ruxton et al. (2018); Summers et al. (2015); White and Umbers (2021) for reviews and discussion), and theoretical modeling supports the conclusion that aposematic species are less constrained by the visual properties of their habitat (habitat generalists) than undefended background-matching species (habitat specialists) (Merilaita and Tullberg 2005; Speed et al. 2010). The appearance of an animal against its visual background fundamentally impacts its detectability by potential predators, a crucial factor shaping the design and function of defensive coloration (see van den Berg, Endler et al. (2023) for discussion). Therefore, assuming relaxed selection for background matching, secondary defenses in a diverse prey community inhabiting visually complex habitats might facilitate chemically defended species to inhabit distinct and possibly more diverse visual backgrounds than undefended species. However, whether the presence of chemical defenses qualitatively or quantitatively correlates with general among-species differences in background habitats remains unknown.

Here, using 12 species of co-occurring Eastern Australian nudibranch species (Fig. 2), we tested the “escape” part of the ‘escape and radiate’ hypothesis by hypothesizing that more defended species inhabit distinct and more variable habitats. Nudibranchs are an intriguing system for the study of defensive animal coloration due to potent chemical defenses in many species (Avila 1995; Winters et al. 2018; Winters et al. 2022) and color patterns ranging from bold aposematic displays to near-perfect camouflage (Debelius and Kuiter 2007). Indeed, the evolution of specialized glands (mantle dermal formations) for the storage and secretion of defensive chemicals (Wägele et al. 2006; Carbone et al. 2013), in combination with the evolution of aposematic signaling, may have led to adaptive radiation in nudibranchs (Gosliner 2001).

Until recently, studies quantifying visual backgrounds in the context of defensive animal coloration have relied on the independent consideration of spatial and spectral (color and luminance) properties (e.g. Cortesi and Cheney 2010; Nokelainen et al. 2021). In this study, we considered these pattern components in combination (van den Berg et al. 2020) using calibrated digital photography, the multispectral image calibration and analysis toolbox (MICA) (Troscianko and Stevens 2015) and its’ integrated quantitative color pattern analysis (QCPA) framework (van den Berg et al. 2020). This methodology enabled us to assess the spatiochromatic information of natural backgrounds upon which nudibranch individuals (12 species, *n* = 184) were found. QCPA provides a descriptive array of image statistics capturing the spatiochromatic properties of each background according to the physiological limitations of an ecologically relevant observer. Here, we used the visual system of a triggerfish (*Rhinecanthus aculeatus*), a common, omnivorous reef fish.

We analyzed different viewing distances to model an escalating predation sequence to account for changes in background appearance to a predator up close (2 cm) and at a distance (30 cm). We then compared these color pattern parameters to measures of the strength of chemical defense for each species, which ranged from highly unpalatable to palatable, using previously published data from antifeedant assays with rockpool shrimp (*Palaemon serenus*) and brine shrimp (Winters et al. 2022). We hypothesized that chemically defended (unpalatable) species would be found on more variable and visually distinct backgrounds, compared with those of undefended (palatable) species. Furthermore, we predicted a strong correlation between the presence and strength of chemical defenses and differences in spatiochromatic properties of background habitats among species with differing strengths of chemical defenses. We expected to find such differences at distances where prey detection and identification are most likely (Endler 1986, 1991; Ruxton et al. 2018).

## Materials and methods

### Study species

**Fig. 1.**
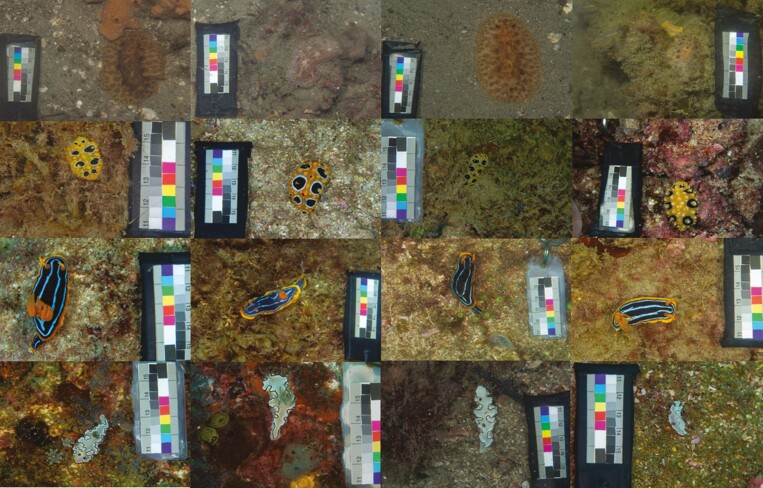
Representative nudibranch and background images for 4 out of the 12 species in this study. From top to bottom: *Discodoris sp., Phyllidia ocellata, Chromodoris kuiteri, Doriprismatica atromarginata*.

**Fig. 2.**
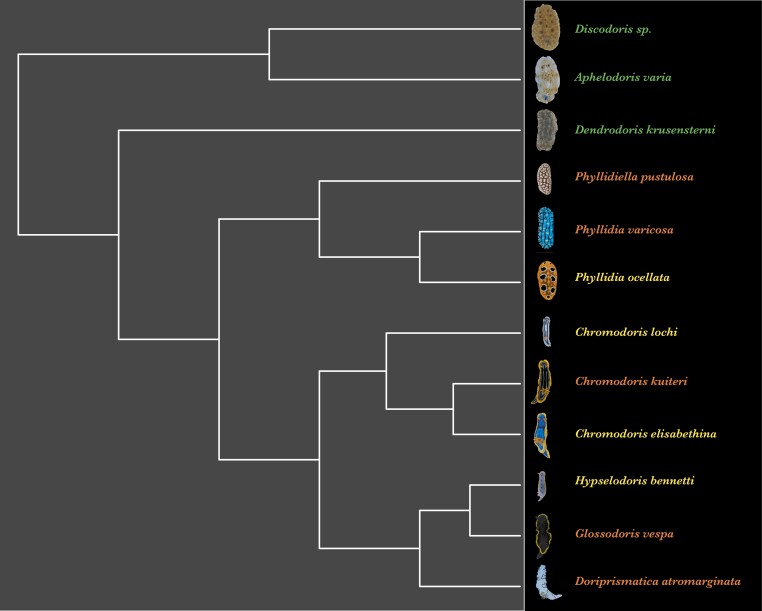
Putative phylogenetic relationships of the 12 species used in this study [modified from Cheney et al. (2014); see methods for details]. Species names are color coded by their category of chemical defenses based on whole-body extract assays with palaemon shrimp to assess unpalatability (1-Effective Dose, ED_50_) and brine shrimp to assess toxicity (1-Lethal Dose, LD_50_) values (Table S1). Green: No known chemical defenses; Yellow: Toxic and moderately unpalatable; Orange: Toxic and highly unpalatable. For a more detailed depiction of species representatives, see Fig. S1.

We took calibrated digital photographs of 184 Dorid nudibranch individuals belonging to 12 species from 4 locations on the east coast of Australia: Sunshine Coast (SE Queensland, Queensland), Gold Coast (SE Queensland), Cook Island (New South Wales, New South Wales) and Nelson Bay (New South Wales) between March 2016 and February 2021 (Table S1, Fig. 2). Our study considers many of the more commonly found Dorid nudibranchs in the study sites (e.g. Larkin et al. 2018; Smith and Davis 2019; Schubert and Smith 2020). Species were identified visually using various nudibranch ID guides (Debelius and Kuiter 2007; Coleman et al. 2015; Gosliner et al. 2018): *Aphelodoris varia* (*n* = 22), *Chromodoris elisabethina* (*n* = 21), *Chromodoris kuiteri* (*n* = 17), *Chromodoris lochi* (*n* = 3), *Dendrodoris krusensterni* (*n* = 7), *Discodoris sp.* (*n* = 13), *Doriprismatica atromarginata* (*n* = 27), *Glossodoris vespa* (*n* = 15); *Hypselodoris bennetti* (*n* = 10); *Phyllidia ocellata* (*n* = 23); *Phyllidia varicosa* (*n* = 8); *Phyllidiella pustulosa* (*n* = 18); numbers indicate sample sizes used in the analysis. *Discodoris sp*. in our study may have constituted a mixture of closely related species, including *Sebadoris fragilis*, *Jorunna pantheris, Tayuva lilacina* and undescribed species; however, these species are visually indiscriminate. Therefore, they are named *Discodoris sp.* as they were found in the same locations, have no known chemical defenses, are predominantly nocturnal and are closely related (Larkin et al. 2018). Only 1 out of the 12 species (*D. atromarginata*) was found in comparable numbers across all sites in NSW and SE QLD, with most species found either in NSW or SE QLD (Table S1). Nudibranchs were photographed underwater against their natural habitat using a calibrated Olympus EPL-5 with a 60 mm macro lens in an Olympus PT-EP10 underwater housing using white LED illumination from a combination of VK6r and PV62 Scubalamp video lights (van den Berg, Troscianko et al. 2020). All pictures were taken at roughly a 90-degree angle relative to each animal and its background, with the background making up roughly 80% of the image (Fig. 1). Nudibranchs generally do not reflect much light below 400 nm (e.g. Cortesi and Cheney 2010) with many potential nonplanctivorous fish predators likely lacking UV vision (Marshall et al. 2003). We therefore used an unmodified camera with spectral sensitivities ranging from 400 nm to 700 nm. For details on camera and image calibration, please see the Supplement.

### Image analysis

Using ImageJ (Schneider et al. 2012) and the MICA toolbox (Troscianko and Stevens 2015), the images were manually segmented into regions of interest (ROI), selecting the animal from its background and defining a size standard using a custom-made color and gray standard placed in each image (Fig. 1). Backgrounds were selected by manually drawing an outline around the background area immediately surrounding each animal, excluding areas out of focus or subject to excessive shadows cast by the artificial illumination. For detailed information on the process of ROI selection and image preparation see the worked examples in the supplement of van den Berg et al. (2024). All images were rotated, with the majority of each animal’s body oriented vertically (head up) before analysis using QCPA (van den Berg, Troscianko et al. 2020). To analyze the images, we used the visual system parameters of a trichromatic triggerfish, *R. aculeatus* (with spectral sensitivities of 413 nm, 480 nm, and 528 nm *λ*_max_; Cheney et al. 2013). This species (adult total length, TL ~ 15 cm) is a typical shallow reef inhabitant found throughout the Indo-Pacific and feeds on invertebrates, algae, and detritus (Randall et al. 1997) and is deemed a potential predator of nudibranchs. We analyzed for viewing distances of 2 cm and 30 cm, assuming a triggerfish spatial acuity of 3 cycles per degree (Champ et al. 2014). The viewing distances reflect a range covering the maximal distance at which the largest specimen and coarse background detail would be detectable (30 cm) to the closest possible distance, reflecting ultimate contact between predator and prey, or visual background (2 cm). Following acuity modeling, the images were processed with a Receptor Noise Limited (RNL) ranked filter (falloff: 3, radius: 5, repetition: 5) to correct for artificial blur introduced by Gaussian filtering. The images were then segmented into color patches discernable to *R. aculeatus* using RNL clustering with a color discrimination threshold of 2∆*S* (Cheney et al. 2019) and a luminance contrast threshold of 4∆*S* (van den Berg et al. 2020) where, ∆*S* corresponds to the Mahalanobis distance between points in the RNL color and luminance space (Vorobyev and Osorio 1998; Kelber et al. 2003; van den Berg et al. 2020). We used Weber fractions based on a relative photoreceptor abundance of 1:2:2:2 (sw:mw:lw:dbl) and photoreceptor noise of 0.05, resulting in 0.07:0.05:0.05:0.05.

QCPA analysis was achieved using a custom batch script (van den Berg et al. 2024), resulting in a highly descriptive array of 157 color pattern statistics for each animal’s visual background. These parameters were spread across the following subtypes of color pattern analysis: (1) color adjacency analysis (CAA). CAA uses a transition matrix tallying all the synonymous and nonsynonymous transitions along horizontal and vertical sampling transects across an image segmented into color pattern elements. This transition matrix is then used to describe the geometry of a color pattern (Endler 2012; van den Berg et al. 2020). (2) Visual contrast analysis (VCA). VCA uses the relative abundance and spectral properties of color pattern elements to describe visual contrast (Endler and Mielke 2005; van den Berg et al. 2020). (3) Boundary strength analysis (BSA). BSA uses the relative abundance of boundaries between color pattern elements to describe visual contrast (Endler et al. 2018; van den Berg et al. 2020). (4) Local edge intensity analysis (LEIA). LEIA quantifies the strength and abundance of edge contrast in an unsegmented image. Statistics ending with “hrz” or “vrt” are the horizontal (across body axis) and vertical (along body axis) versions (analyzing the respective transition matrix only) of their respective statistic (analyzing the full transition matrix). For example, a background with algae or seagrass will likely have an elongated aspect ratio, whereas a uniform sandy background will not. A detailed description of each pattern statistic can be found in van den Berg et al. (2020). A simplified summary of all parameters and their abbreviations can be found in Table S2.

### Level of chemical defense

As a measure of chemical defense for each species, we used previously published data from antifeedant assays with rockpool shrimp (*Palaemon serenus*) and toxicity assays with brine shrimp (Winters et al. 2022). Assay data for *Glossodoris vespa* is presented in Winters et al. (2018). In summary, the data were obtained from extracting secondary metabolites from nudibranchs and added to food pellets made from squid mantle at increasing concentrations. Effective dose (ED_50_) values were calculated based on the concentration that elicited a rejection response in at least 50% of the rockpool shrimp. Lethal dose (LD_50_) values were calculated based on the concentration that killed at least 50% of brine shrimp. For this study, the resulting ED_50_ and LD_50_ values were normalized and calculated as 1 – ED_50_/1 – LD_50_ to range from 0 (most palatable/toxic) to 1 (least palatable/toxic). Where multiple estimates of ED_50_ existed for a species due to multiple extracts/assays being performed, the average value was used. Only assays using whole-body extracts were considered to allow for comparisons between species (Table S1).

To ensure at least 3 species in each group of chemical defenses (*n* = 3–5, Fig. 2) and to allow for subsequent investigations of differences in backgrounds between species with different levels of chemical defenses, we separated the species into those who had no known toxicity and unpalatability, those with some level of toxicity and moderate unpalatability and those with some level of toxicity and high unpalatability (Fig. 2 and Table S1). Due to the uneven spread of toxicity and palatability values among species, we classified each species according to the shape of a sigmoidal response curve where species with values of 0 were considered palatable, values up to 0.25 were considered weakly unpalatable, values between 0.25 and 0.74 medium unpalatable and values of 0.74 and higher as highly unpalatable. We chose 0.74 rather than 0.75 as the boundary between medium and high unpalatability, as this was the median unpalatability of species with chemical defenses (Table S1).

### Statistical analysis

To analyze the large dataset derived from the QCPA analysis, we only kept images that did not produce any missing value for any background pattern metrics. VCA, CAA, and BSA metrics can produce NaN or infinite values if a color pattern has less than 2 color pattern elements following RNL clustering. While this approach removes extremely homogenous backgrounds (excluded images: 2 cm *n* = 2, 30 cm *n* = 33), with more NaNs appearing at greater viewing distances due to spatial downsampling, it ensures using the largest possible color pattern space. Thus, our analysis excludes uniform backgrounds, i.e. those without any color patterning due to differences in luminance or color beyond the perceptual thresholds defined for image segmentation.

We then applied a latent variable Exploratory Factor Analysis (EFA) to identify latent variables best describing differences between our 3 groups of variably defended species in our 157-dimensional colorspace, thus reducing its dimensionality. This was done with the R package *psych* using the factoring method of Ordinary Least Squares “ols” and the orthogonal rotation “varimax.” To prepare the dataset for the EFA, we first filtered the number of highly correlated QCPA metrics by keeping those that were less correlated than 0.6 Pearson correlation, which reduced their number from 157 to 17. We then ran the factor analysis based on 4 factors. The number of factors was selected by comparing the eigenvalues calculated from the original dataset to the median eigenvalues extracted from 10,000 randomly generated datasets with the same rows and columns as the original data. We selected factors with eigenvalues greater than the median of the eigenvalues from the simulated data. We also computed bootstrapped confidence intervals of the loadings by iterating the factor analysis 1,000 times.

Due to data filtering for metrics less correlated than 0.6, the QCPA parameter listed for a given loading is likely synonymous with various other parameters in our 157-dimension color pattern space. Therefore, the precise wording to describe each factor can vary substantially depending on which color pattern metrics are put into focus (for a complete list of parameter correlations, see Table S3).

The scores of the factors extracted from the EFA were then used to implement 4 phylogenetic, distributional linear mixed models to compare the natural backgrounds of nudibranchs with different levels of chemical defenses. Models were run in R v 4.1.2 (R Core Team 2021) using the brms package (Bürkner 2018), which fits Bayesian models using Stan (Stan Development Team 2024). To account for the phylogenetic dependency of closely related species, all models included the phylogenetic tree of the 12 species of nudibranchs (Fig. 2), with the tree from Cheney et al. (2014) pruned and missing species added according to their taxonomic classification in the World Register of Marine Species (Bernot et al. 2024). The phylogenetic model was implemented following the guidelines of the online brms vignette (https://cran.rproject.org/web/packages/brms/vignettes/brms_phylogenetics.html) based on de Villemereuil and Nakagawa (2014).

The first model investigated differences in scores for latent factor 1 between nudibranchs with different levels of chemical defenses (see chemical defenses section) using a Student distribution. The model estimated the effect of the main categorial predictors level of chemical defense (low, moderate, and high) and observer distance (2 cm and 30 cm) and their interaction on the response distribution’s mean and the residual standard deviation. To account for repeated measurements of each species, we also included species ID as a random intercept to the model. We further included random slopes over distance because their relationship with the value of the response factor 1 changes among species.

The second, third, and fourth models were identical to the first but used factor 2, factor 3, and factor 4 as response variables. All models were fitted using weakly informative prior distributions (normal with mean = 0 and SD = 5 for intercept and coefficients, exponential (1) for standard deviations). Their performance was evaluated using posterior predictive model checking, which compares model predictions with observed data to assess overall model fit. We ran 4 Markov–Chain–Monte–Carlo (MCMC) chains for each model and obtained coefficient estimates from 8,000 post-warm-up samples. All model parameters reached reliable conversion indicators (Korner-Nievergelt et al. 2015; Santon et al. 2023): A Monte Carlo standard error smaller than 5% of the posterior standard deviation, an effective posterior sample size greater than 10% of the total sample size, and a $\hat{R}$ statistic value smaller than 1.01.

For graphical displays of the results, we present—for each combination of chemical defenses and distance—the medians of latent factors values and their 95% credible intervals (CIs) of the posterior distributions of fitted values for the population average obtained from the joint posterior distributions of the model parameters (Korner-Nievergelt et al. 2015). The same posterior distribution of fitted values was used to compute pairwise differences and their 95% CIs between all possible combinations of the same 2 categorical predictors using the emmeans R package (Lenth 2023). To compare the variance of response values between all combinations of predictor levels, we also compute the posterior distribution of all pairwise differences of the residual standard deviation on the original scale (back-transformed from the log scale). The effect size of pairwise differences increases with increasing deviation of such differences from zero, and the robustness of the result increases with decreasing degree of overlap of the 95% CI with zero. For a simplified schematic of the methodology, see Fig. S2.

## Results

**Fig. 3.**
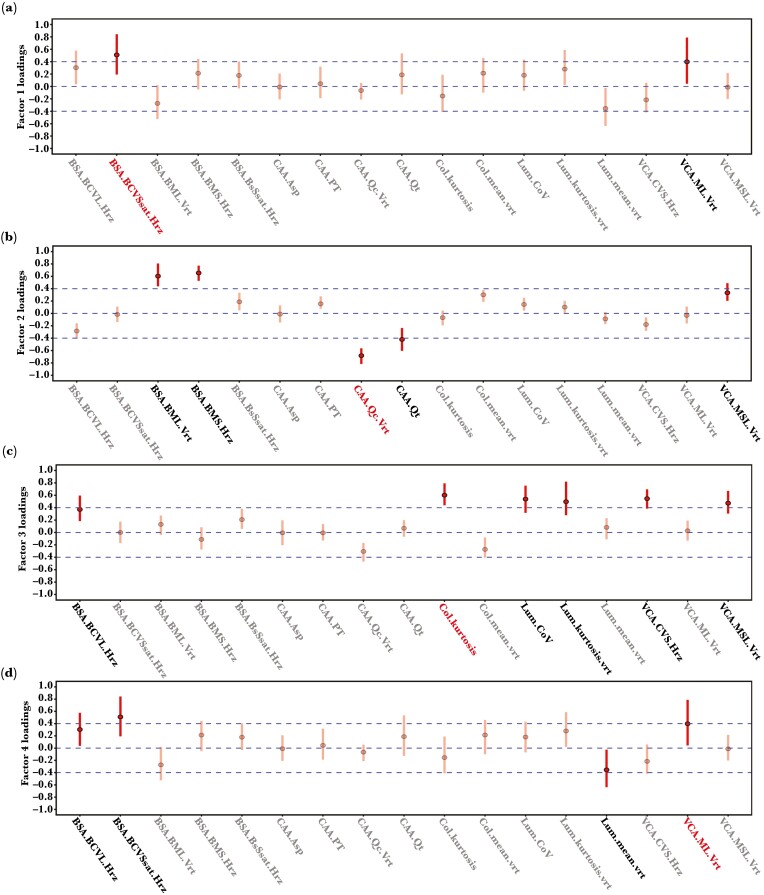
Detailed visual representation of the loadings of each factor (a–d). (a) Factor 1; (b) Factor 2; (c) Factor 3; (e) Factor 4. Greyed-out factor loadings indicate color pattern descriptors with minor contributions (<0.4) to each factor. Parameters indicated with bold red writing are represented visually with their respective minimum and maximum value background in the dataset (Fig. S5).

Using the EFA, we identified 4 factors to describe how background pattern metrics differ between multiple viewing distances and levels of chemical defenses. While not intended to identify a maximal amount of variability in color pattern variation in our dataset, the 4 factors still explain 39% of the total variation (factor 1: 11%; factor 2: 11%, factor 3: 11%, and factor 4: 6%) (Fig. 3). Looking at the loadings of each factor, we can identify what latent variable they describe. While it would be possible to discuss each factor in almost infinite depth, we keep their description to loadings of ±0.4 to capture their main properties. For visual examples of various parameters with high loadings see Fig. S3. For visual examples of backgrounds with the highest and lowest value of each factor see Fig. S4.

### Factor 1: Higher luminance and chromatic contrast coincides with smaller patch size

Factor 1 (Fig. 3a) describes 11% of the variation in visual backgrounds and captures a positive relationship between increases in the mean RNL luminance edge contrast (e.g. *Lum.mean*) and RNL chromaticity edge contrast (e.g. *Col.mean*) as measured by LEIA (unclustered image) and the average size of color pattern elements in an RNL clustered image (*CAA.PT*). The increase in average luminance and chromatic edge contrast in visual backgrounds also coincides with an increase in variability of the luminance contrast relative to the mean (e.g. *BSA.BCVL*).

**Fig. 4.**
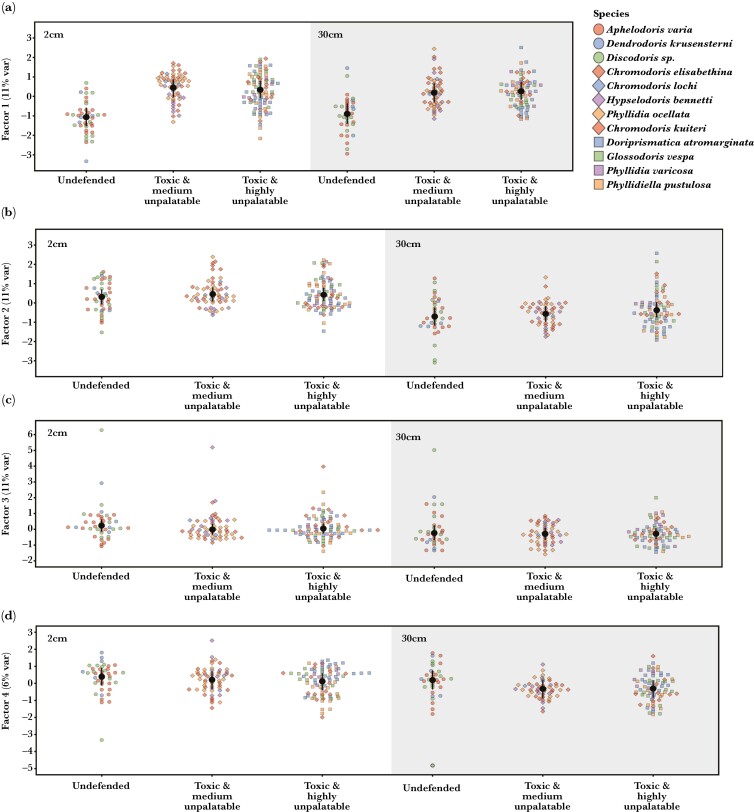
Factor value estimates for each group (a–d) and viewing distance (left vs. right side panel). (a) Factor 1; (b) Factor 2; (c) Factor 3; (d) Factor 4.

The visual backgrounds of chemically defended species have noticeably higher levels of factor 1 than those of undefended species [difference (±95% CI)] (Fig. 4a). This is true up close (2 cm) as well as from further away (30ccm) for toxic species with moderate levels of unpalatability (2 cm: –1.53 (–2.28/–0.78); 30 cm: –1.05 (–1.83/–0.23)) as well as toxic species with high levels of unpalatability (2 cm: –1.44 (–2.18/–0.73); 30 cm –1.17 (–1.92/–0.37)). However, toxic species with moderate levels of unpalatability have similar factor values to species with high levels of unpalatability up close (2 cm: 0.08 (–0.38/0.54); 30 cm: –0.13 (–0.63/0.36)). Therefore, chemically defended species were found on visual backgrounds with more chromatic and achromatic contrast (Table S4). However, the variability of backgrounds on which species were found did not differ between groups and remained similar at 2 cm and 30 cm (Table S5).

### Factor 2: Higher color and luminance contrast between patches coincide with lower background homogeneity

Factor 2 (Fig. 3b) describes 11% of the variation in visual backgrounds and mostly captures the correlation between simultaneous increases in the strength of luminance contrast (e.g. *BSA.BML*, *V*CA.*MSL*) and saturation contrast (e.g. *BSA.BMS*) between color pattern elements and decreases in background evenness (e.g. *CAA.Qt* & *CAA.Qc*).

We found no differences in factor values between species with different levels of chemical defenses [difference (±95% CI)] (Fig. 4b and Table S6) nor any differences between groups in the variability of factor values (Table S6) at 2 cm or 30 cm viewing distance. All groups showed higher factor values at 2 cm compared with 30 cm (Fig. 2b, undefended: 1.01 (0.57/1.48); toxic moderately unpalatable: 1.01 (0.67/1.31); toxic highly unpalatable: 0.80 (0.47/1.11)). The variability of backgrounds on which species were found did not differ between groups and remained similar at 2 cm and 30 cm (Table S7).

### Factor 3: Increased luminance and color contrast variability correlate with reduced average color pattern regularity

Factor 3 (Fig. 3c) captures the relationship between increases in the variability of luminance contrast in visual backgrounds (e.g. *Lum.kurtosis*, *Lum.CoV*) as well as the variability of color contrast (e.g. *Col.kurtosis*, *V*CA.*CVS*) and a decrease of the average color contrast (e.g. *Col.mean*) as well as color pattern regularity (e.g. *CAA.Qc*).

We found no differences in factor values of backgrounds between groups with different levels of chemical defenses [difference (±95% CI)] (Fig. 4c and Table S8) at either 2 cm or 30 cm. However, all groups showed increased factor values at 2 cm compared with 30 cm (Fig 2c; undefended: (0.50 (0.07/0.91)); toxic moderate unpalatable: toxic highly unpalatable species (0.34 (0.08/0.59)). No difference in the variability of factor values was detected between the background of groups at either viewing distance, and background variability was the same at 2 cm and 30 cm for all groups (Table S9).

### Factor 4: Increases in achromatic patch contrast correlate with decreases in achromatic boundary contrast, but increases in chromatic boundary contrast variability

Factor 4 (Fig. 3d) describes a correlation between achromatic average patch contrast (e.g. *V*CA.*ML*) and measures of achromatic boundary contrast (e.g. *Lum.mean*, *BSA.BML*). Specifically, increases in mean luminance contrast relative to the size of color pattern elements coincide with a decrease in the average luminance contrast of boundaries between color pattern elements. Furthermore, decreases in the mean luminance contrast of pattern boundaries correlate with an increase in chromatic boundary contrast variability relative to the mean (e.g. *BSA.BCVSsat*).

We did not find any differences in factor values between the backgrounds of species with different levels of chemical defenses at either 2 cm or 30 cm [difference (±95% CI)] (Fig. 4d, Table S10). However, while undefended species showed no difference in factor values of backgrounds between viewing distances (0.21 (–0.25/0.69)), both toxic and medium unpalatable species (0.53 (0.25/0.81)), as well as toxic and highly unpalatable species (0.42 (0.16/0.70)), had higher factor values for their visual backgrounds at 2 cm compared with 30 cm. No differences in background variability between groups were detected at either viewing distance (Table S11), except for undefended species having more variable backgrounds at 30 cm compared with toxic and moderately defended species (0.45 (0.07/0.90)). The variability of visual backgrounds did not differ between viewing distances for either group (Table S11).

## Discussion

Our analysis captured 4 latent variables describing correlations between the spatiochromatic properties of visual backgrounds on which animals from 12 species of nudibranch molluscs were found (Fig. 3). In agreement with 1 of our predictions derived from the “escape and radiate” hypothesis, these latent variables showed that nudibranch species with chemical defenses, irrespective of their relative strength, were found on visual backgrounds distinct in their appearance from backgrounds of undefended species according to the physiological limitations of a potential predator, a triggerfish (*R. aculeatus*) (Fig. 4a). However, while some visual properties of backgrounds varied significantly more up close (2 cm) than from further away (30 cm), we did not find any differences in the variability of visual backgrounds between species with different levels of chemical defenses. This lack of among-species variability indicates that chemical defenses and their relative strength do not correspond to increase among-species background variability in our dataset, contrary to the predictions derived from the “escape and radiate” hypothesis (Thompson 1989; Merilaita and Tullberg 2005). Instead, we suggest that chemical defenses in Dorid nudibranchs coincide with broad, yet equally variable, differences in visual background habitats.

As shown by the composition of each factor (Fig. 3), multicomponent descriptors of complex phenotypes can be difficult to reduce to a low-dimensional, intuitive, spatiochromatic property that adequately captures the underlying complexity and links it to perceptual and functional properties of coloration. Unlike numerical classifiers from artificial neuronal networks or other machine learning approaches used in high-dimensional image analyses for computer vision (e.g. Serre et al. 2007; Talas et al. 2019), each parameter in our color pattern space describes a specific spatiochromatic property (van den Berg et al. 2020). Therefore, despite a range of complexity in associations with response variables (see van den Berg et al. (2022) for discussion), we can make assumptions about the perceptual processes associated with our latent predictors.

Visual backgrounds of chemically defended species in our study can be broadly characterized by the presence of increased color and luminance contrast between objects and surfaces when compared with visual backgrounds of undefended species (Fig. 4a). This difference in the visual appearance of background habitats to a potential predator was equally as strong when viewed from 2 cm than at 30 cm. Therefore, backgrounds of chemically defended species remained more spatiochromatically contrasting than those of undefended species (Fig. 4a), even at distances where substantial amounts of spatial information would be lost to a potential predator. This persistence of spatiochromatic variability in visual backgrounds across viewing distances is of interest in the context of distance-dependent selection pressures shaping the ecology and evolution of color pattern diversity in prey communities (e.g. Endler 1978; Barnett et al. 2016; van den Berg et al. 2022; van den Berg, Endler et al. 2023). For example, imperfect mimicry among and color pattern polymorphism within aposematic species could be shaped by their adaptive value in the context of predator perception at multiple viewing distances. Persisting variability of visual backgrounds across viewing distances could be selecting for perceptual similarities between animals and backgrounds in a more general way than previously considered.

Aposematism is assumed to be widespread in Dorid nudibranchs (Rudman 1991), with bold colors and patterns coinciding with the presence of chemical defenses (Cortesi and Cheney 2010; Winters et al. 2018; Winters et al. 2022). However, the coincidence of secondary defenses and boldly contrasting animal coloration (e.g. van den Berg, Endler et al. 2023) does not mean that the colors and patterns displayed by a species necessarily serve a warning function (see Summers et al. 2015; White and Umbers 2021) for review). Instead, the function of bold coloration in an environment with increased color and luminance contrast is likely complex and might even assist in camouflage (Endler 1978; Marshall and Stevens 2014; van den Berg, Endler et al. 2023). The detectability of an aposematic animal is determined by its appearance against its visual background and is likely fine-tuned to the cost–benefit trade-offs of increased predator encounters (e.g. Barnett et al. 2016; van den Berg, Endler et al. 2023). Therefore, assuming substantial (yet variable) degrees of signaling honesty in Dorid nudibranchs (e.g. Cortesi and Cheney 2010), our study suggests broadly generalizable correlations between the presence of chemical defenses in aposematic species and the spatiochromatic properties of their visual habitat. However, these constraints can be variably explained by the need for efficient camouflage (e.g. Speed et al. 2010; Barnett et al. 2016) or signaling efficacy (e.g. Endler and Mappes 2004; Speed et al. 2010) at variable viewing distances. How specific spatiochromatic properties of visual backgrounds highlighted by our study impact the signaling function of aposematic coloration and camouflage in the considered species would be of great interest in future behavioral experimentation studies.

Nudibranchs mainly use chemotaxis to move around their environment to find food and mates, with visual input only relevant for basic phototaxis, such as determining daytime or detecting shelter (Eakin et al. 1967). Therefore, unlike other aposematic species, such as insects or frogs (Higginson et al. 2012; Rojas 2017), nudibranchs will unlikely choose resting and foraging microhabitats based on visual cues. Therefore, it is likely that the backgrounds on which species are found are the indirect rather than direct consequence of correlations between their habitat’s visual appearance and other sensory modes and selective pressures shaping the efficacy of visual defenses. Thus, finding chemically defended species on distinct visual backgrounds fits well with assumed radiation in feeding ecology, enabling the acquisition of secondary defenses (e.g. Winters et al. 2018).

To our knowledge, no study has quantified the mobility and the diets of many nudibranch species (but see Rudman and Bergquist 2007 for feeding specificity in chromodorid nudibranchs), and the spatiotemporal distribution of food sources remains poorly known. Some of the most unpalatable nudibranchs in this study are known to forage on sponges containing highly potent secondary metabolites (e.g. latrunculin a) at least at some stage during their lifetime (e.g. *C. elisabethina*) (Cheney et al. 2016). In contrast, others can synthesize compounds de novo (Cimino and Ghiselin 1999). Nudibranchs with more specialist diets may need to increase mobility to find uncommon food sources. The sponge diet consequently allows the animals to maintain functional levels of secondary metabolites or pigments required to maintain salient aposematic color patterns. Background specialization might, therefore, be increased in species dependent on such supplemental food sources and could result in variable degrees of within and among-species background variability. For example, astonishing cases of prey masquerade are common among nudibranch species (e.g. in the genus *Phylodesmium* (Burghardt et al. 2008)). Thus, the assumption that increased background variability indeed corresponds to an increase in niche space may or may not be appropriate for a given species of nudibranchs (Arbuckle et al. 2013). However, as our study focuses on the visual appearance of backgrounds only, whether the presence of secondary defenses in the context of visual background habitats also coincides with changes in defensive animal coloration remains an exciting and crucial avenue for future research.

The mechanisms underlying the “escape and radiate” hypothesis are often unclear, with little empirical evidence of the process (e.g. Suchan & Alvarez 2015). For example, evidence of increased background variability could only be present during certain stages of (rapid) speciation in aposematic animals. More extensive sampling of primary and secondary defenses and corresponding visual backgrounds within and among species, considering each species’ geographic distribution and phenotypic variability, would be of great interest for future research. Combined with an increased resolution of existing heterobranch phylogenies (e.g. Layton et al. 2018, 2020), this would enable a more detailed investigation into the presence and scale of animal background and color pattern diversification and its correlation with secondary defenses. For example, the storage of modified secondary metabolites and the evolution of dedicated body parts for their storage (such as mantle dermal formations, MDFs) has been suggested to be a derived trait among nudibranchs (Gosliner 2001; Cheney et al. 2016). There is some evidence that suggests that camouflage is an ancestral trait among heterobranch sea slugs, rather than aposematism (Cortesi and Cheney 2010). Indeed, increased sample sizes would enable species, rather than group-based analyses such as the one presented in this study. Furthermore, the imputation of missing values (Johnson et al. 2021) in future analyses might provide an avenue for maximizing the dimensionality of color pattern space whereas minimizing the need to exclude observations. However, how and if such approaches are suitable for the type of data presented in our study remains to be seen.

Despite some of the discussed limitations of data available to the study of defensive coloration in nudibranchs, there are crucial advantages over more established (predominantly terrestrial) systems such as insects, amphibians or even mammals (see Kikuchi et al. 2023; Ruxton et al. 2018 for review). Heterobranch sea slugs are hermaphrodites, possess rudimentary vision (e.g. Eakin et al. 1967) and are unlikely to rely on thermoregulation by absorbing heat from sunlight. This significant reduction in coinciding selective pressures greatly aids in attributing observed correlations between visual background diversity and chemical defenses (and subsequent conclusions) to natural selection by visual predation. The direction, speed and extent by which natural selection would constrain correlations between the strength of secondary defenses and background habitat are likely determined by multiple, currently poorly understood factors such as the fitness benefits of specific color patterns across different visual backgrounds, the diversity of visual backgrounds inhabited by a given species, the protective value of secondary compounds across complex predator communities and the heritability of visual phenotypes, to name a few. For example, background diversity is likely to also favor visual defenses such as disruptive coloration (e.g. Endler 2006) and compromise camouflage (e.g. Houston et al. 2007) which could be used variably and coincidingly with general background matching in nudibranchs (e.g. Bulmer 1972; Endler 1978; van den Berg, Endler et al. 2023). Nevertheless, our study provides empirical support to the possibility of secondary defenses coinciding with generalizable differences in visual background habitats in a complex community of nudibranch molluscs. We demonstrate this by employing sophisticated methodology aimed at reflecting the visual processing of ecologically relevant observers and utilizing a high-dimensional, highly differentiated approach to quantifying spatiochromatic properties of visual backgrounds.

## Supplementary Material

arae053_suppl_Supplementary_Material

## Data Availability

Analyses reported in this article can be reproduced using the data provided by the authors (van den Berg, Santon, et al. 2023).
