# Supplementary material for: Highly defended nudibranchs “escape” to visually distinct background habitats
Source: Behav Ecol. 2024 Jul 4;35(5):arae053. doi: 10.1093/beheco/arae053 (PMC11289952; doi:10.1093/beheco/arae053)
Supplement: arae053_suppl_Supplementary_Material [file arae053_suppl_supplementary_material.pdf]

Additional information on camera calibration & image standardisation

The detailed method applied for camera calibration and associated camera spectral sensitivity can be found here:

https://www.empiricalimaging.com/knowledge-base/make-your-own-camera-callibration/

The camera spectral sensitivity is freely available as part of the micaToolbox, which can be downloaded here:

https://www.empiricalimaging.com/download/micatoolbox/

For details of the illuminant used in the analysis and a detailed step by step guide of the image analysis used for this study, please refer to the worked example no. 1 in van den Berg et al. 2024.

To access the spectral sensitivity file, go to:

ImageJ → Plugins → Cone Mapping → Cameras → Olympus PEN E-PL5 Olympus 60mm 400-700

For details on the resin cast colour standard used in the study, please see:

https://www.empiricalimaging.com/knowledge-base/make-your-own-colour-grey-standard/ (first example)

The white standard reflectance values used in the image analysis were determined by referencing the respectively used grey value to a 99% spectralon white standard:

	R	G	B
**Std 1:**	**70.027428300**	**66.130511506**	**59.281968855**
**Std 2:**	**84.428484622**	**82.347847979**	**78.229292505**
**Std 3:**	**83.691163421**	**83.262483001**	**81.646165071**

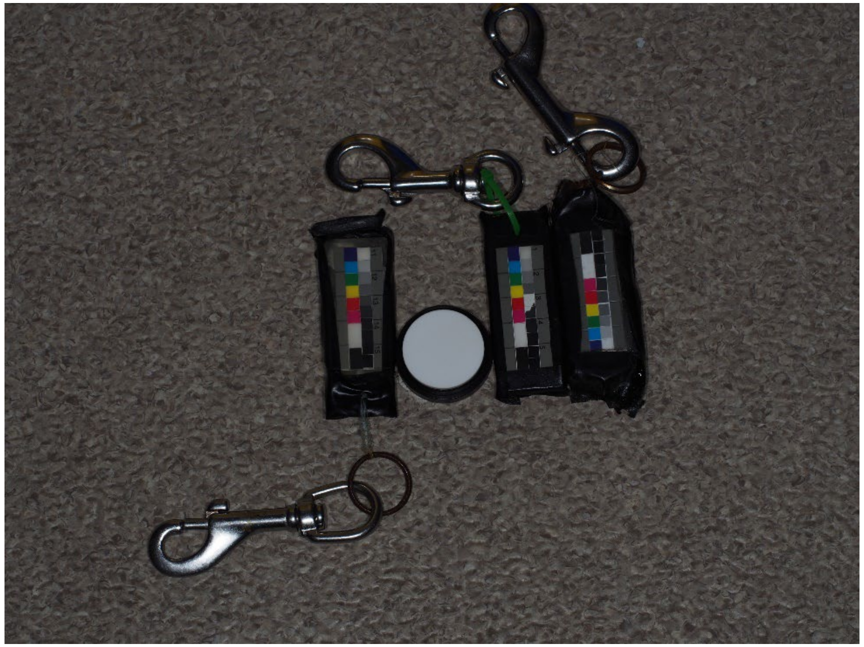

Image of the three colour standards used in the data set. RGB values (0-1) correspond to the largest white patch in each standard relative to the spectralon 99% standard. Standards 1-3 are presented left to right.

Other details:

All images were taken in RAW at ISO 200 with variable aperture and shutter speed to achieve optimal exposure and focus (avoiding overexposure of the white standard).

Table S1

Summary table of all individuals in the dataset used for this study.

Species	Unpalatability (1-ED$_{50}$)	Toxicity (1-LD$_{50}$)	Sampling site			
			Sunshine Coast, QLD	Gold Coast, QLD	Nelson Bay, NSW	Grand Total
Aphelodoris varia	0	0	0	0	22	22
Chromodoris elisabethina	0.55	1	21	0	0	21
Chromodoris kuiteri	0.74	1	10	7	0	17
Chromodoris lochi	0.61	1	3	0	0	3
Dendrodoris krusensterni	0	0	0	2	5	7
Discodoris sp.	0	0	0	0	13	13
Doriprismatica atromarginata	0.74	0.93	16	0	11	27
Glossodoris vespa	0.87	0.29	15	0	0	15
Hypselodoris bennetti	0.73	0.88	0	0	10	10
Phyllidia ocellata	0.63	0.4	23	0	0	23
Phyllidia varicosa	0.92	0.53	8	0	0	8
Phyllidiella pustulosa	0.86	0.14	18	0	0	18
						184

Figure S1

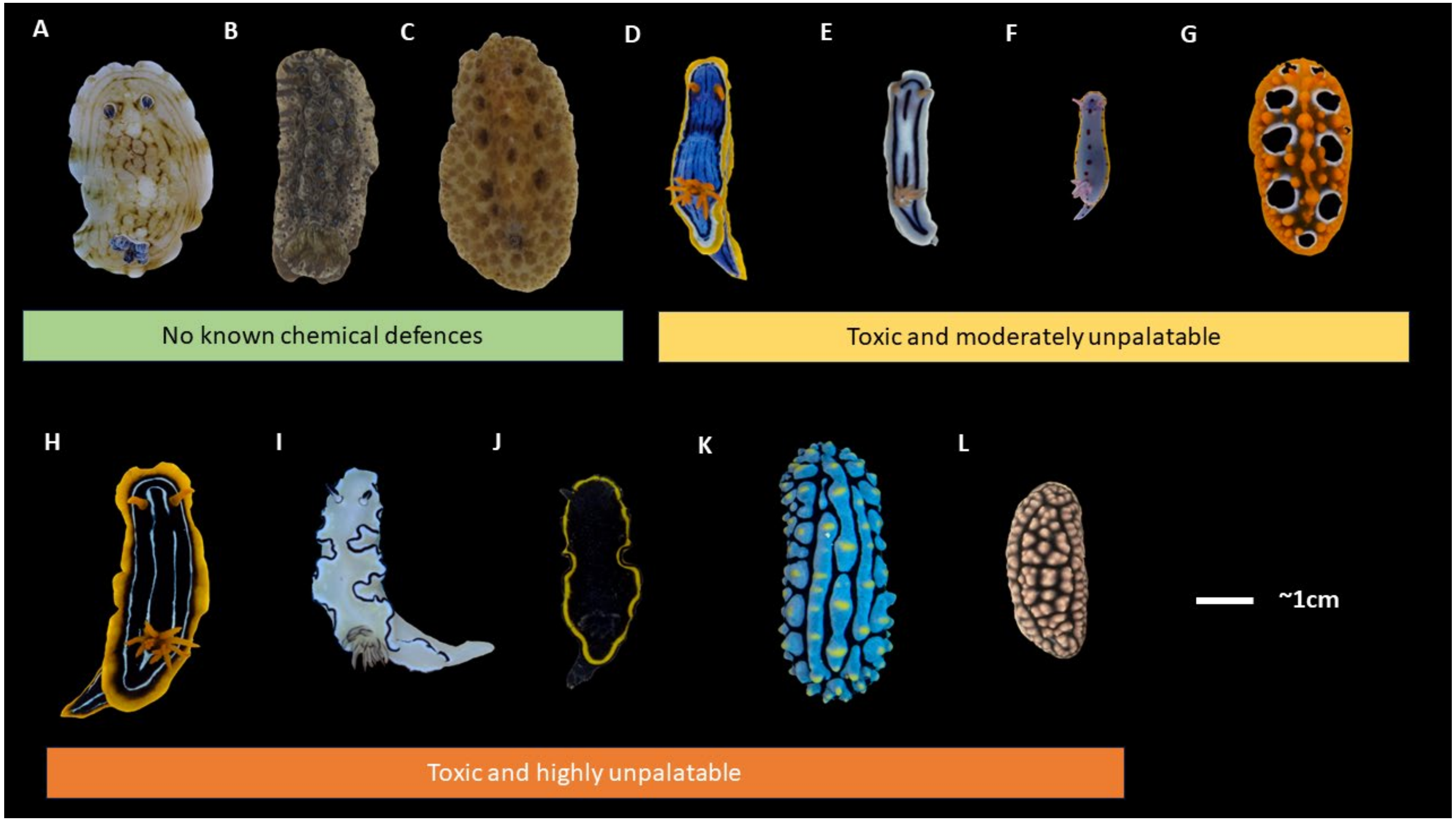

Figure S1. Representative photographs of the 12 species used in this study grouped into categories of chemical defences based on whole-body extract assays with palaemon shrimp to assess unpalatability (1-Effective Dose, ED_{50}) and brine shrimp to assess toxicity (1-Lethal Dose, LD_{50}) values: A) *Aphelodoris varia;* B) *Dendrodoris krusensterni*; C) *Discodoris sp*; D) *Chromodoris elisabethina*; E) *Chromodoris lochi;* F) *Hypselodoris bennetti;* G) *Phyllidia ocellata*; H) *Chromodoris kuiteri*; I) *Doriprismatica atromarginata;* J) *Glossodoris vespa*; K) *Phyllidia varicosa;* L) *Phyllidiella pustulosa.*

Table S2

List of all QCPA colour pattern metrics of the colour adjacency (CAA, yellow), visual contrast (VCA, blue) and boundary strength analysis (BSA, red), with added listing of local edge analysis (LEIA, green) parameters. Modified from van den Berg et al. 2020. For detailed explanations of each parameter and those of LEIA, please see the original publication and associated online documentation.

Variable Name	Abbreviation
Simpson colour diversity - S_c	CAA:Sc
Relative Simpson colour diversity - J_c	CAA:Jc
Simpson transition diversity - S_t	CAA:St
Relative Simpson transition diversity - J_t	CAA:Jt
Shannon colour diversity - H_c	CAA:Hc
Relative Shannon colour diversity - *Qc*	CAA:Qc
Shannon transition diversity - H_t	CAA:Ht
Relative Shannon transition diversity - Q_t	CAA:Qt
Simpson colour pattern complexity S_{cpl}	CAA:Scpl
Shannon colour pattern complexity Q_{cpl}	CAA:Qcpl
Pattern Complexity - *C*	CAA:C
Average patch size – *PT*	CAA:PT

Average horizontal patch size - PT_{Hrz}	CAA:PT Hrz
Average vertical patch size - PT_{Vrt}	CAA:PT Vrt
Aspect ratio – A	CAA:Asp
Weighted mean of pattern **luminance** contrast - M_L	VCA:ML
Weighted standard deviation of pattern **luminance** contrast - s_L	VCA:sL
Weighted CoV of pattern **luminance** contrast - CV_L	VCA:CVL
Weighted mean of pattern ***Dmax*** contrast - M_{Dmax}	VCA:MDmax
Weighted standard deviation of pattern ***Dmax*** contrast - s_{Dmax}	VCA:sDmax
Weighted CoV of pattern ***Dmax*** contrast - CV_{Dmax}	VCA:CVDmax
Weighted mean of pattern **RNL saturation** contrast - ΔS_{Sat}	VCA:MSsat
Weighted standard deviation of pattern **RNL saturation** contrast - $s_{\Delta S_{Sat}}$	VCA:sSsat
Weighted CoV of pattern **RNL saturation** - $CV_{\Delta S_{Sat}}$	VCA:CVSsat
Weighted mean of **RNL luminance** pattern contrast - $M_{\Delta S_L}$	VCA:MSL
Weighted standard deviation of **RNL luminance** pattern contrast - $s_{\Delta S_L}$	VCA:sSL
Weighted CoV of **RNL luminance** pattern contrast - $CV_{\Delta S_L}$	VCA:CVSL
Weighted mean of pattern **RNL chromaticity** contrast - $M_{\Delta S}$	VCA:MS
Weighted standard deviation of pattern **RNL chromaticity** contrast - $s_{\Delta S}$	VCA:sS

Weighted CoV of pattern **RNL chromaticity** contrast - $CV_{\Delta S}$	VCA:CVS
Weighted mean of **luminance** boundary strength - BM_L	BSA:BML
Weighted standard deviation of **luminance** boundary strength - Bs_L	BSA:BsL
Weighted CoV of **luminance** boundary strength - BCV_L	BSA:BCVL
Weighted mean of ***Dmax*** boundary strength - BM_{Dmax}	BSA:BMDmax
Weighted standard deviation of ***Dmax*** boundary strength - Bs_{Dmax}	BSA:BsDmax
Weighted CoV of ***Dmax*** boundary strength - BCV_{Dmax}	BSA:BCVDmax
Weighted mean of **RNL saturation** boundary strength - $BM_{\Delta S_{Sat}}$	BSA:BMSsat
Weighted standard deviation of **RNL saturation** boundary strength - $Bs_{\Delta S_{Sat}}$	BSA:BsSsat
Weighted CoV of **RNL saturation** boundary strength - $BCV_{\Delta S_{Sat}}$	BSA:BCVSsat
Weighted mean of **RNL luminance** boundary strength - $BM_{\Delta S_L}$	BSA:BMSL
Weighted standard deviation of **RNL luminance** boundary strength - $Bs_{\Delta S_L}$	BSA:BsSL
Weighted CoV of **RNL luminance** boundary strength - $BCV_{\Delta S_L}$	BSA:BCVSL
Weighted mean of **RNL chromaticity** boundary strength - $BM_{\Delta S}$	BSA:BMS
Weighted standard deviation of **RNL chromaticity** boundary strength - $Bs_{\Delta S}$	BSA:BsS
Weighted CoV of **RNL chromaticity** boundary strength - $BCV_{\Delta S}$	BSA:BCVS
CoV of the **chromatic RNL LEIA edge distribution**	Col.CoV

Kurtosis of the **chromatic RNL LEIA edge distribution**	Col.kurtosis
Mean of the **chromatic RNL LEIA edge distribution**	Col.mean
Standard deviation of the **chromatic RNL LEIA edge distribution**	Col.sd
Skew of the **chromatic RNL LEIA edge distribution**	Col.skew
CoV of the **achromatic RNL LEIA edge distribution**	Lum.CoV
Kurtosis of the **achromatic RNL LEIA edge distribution**	Lum.kurtosis
Mean of the **achromatic RNL LEIA edge distribution**	Lum.mean
Standard deviation of the **achromatic RNL LEIA edge distribution**	Lum.sd
Skew of the **achromatic RNL LEIA edge distribution**	Lum.skew

Table S3

Summary table of all >.6 Pearson correlation values between parameters with >0.4 loadings for each factor.

FACTOR 1			FACTOR 2			FACTOR 3			FACTOR 4		
**Factor loading >0.4**	**Correlated Param. (>.6)**	**Cor**	**Factor loading >0.4**	**Correlated Param. (>.6)**	**Cor**	**Factor loading of >0.4**	**Correlated Param. (>.6)**	**Cor**	**Factor loading of >0.4**	**Correlated Param. (>.6)**	**Cor**
BSA.BCVSsat.Hrz	BSA.BCVDmax	0.80	BSA.BML.Vrt	BSA.BML	1.00	BSA.BCVL.Hrz	CAA.St	0.61	BSA.BCVL.Hrz	CAA.St	0.61
	BSA.BCVSsat	1.00		BSA.BMSL	1.00		CAA.Ht	0.68		CAA.Ht	0.68
	BSA.BCVDmax.Hrz	0.80		BSA.BML.Hrz	1.00		CAA.St.Hrz	0.62		CAA.St.Hrz	0.62
	BSA.BCVDmax.Vrt	0.79		BSA.BMSL.Hrz	0.99		CAA.St.Vrt	0.61		CAA.St.Vrt	0.61
	BSA.BCVSsat.Vrt	1.00		BSA.BMSL.Vrt	1.00		CAA.Ht.Hrz	0.68		CAA.Ht.Hrz	0.68
VCA.ML.Vrt	VCA.ML	1.00	BSA.BMS.Hrz	BSA.BMS	1.00		CAA.Ht.Vrt	0.68		CAA.Ht.Vrt	0.68
	VCA.sL	0.67		BSA.BMS.Vrt	1.00		CAA.Qt.Hrz	0.63		CAA.Qt.Hrz	0.63
	VCA.MDmax	0.71	CAA.Qc.Vrt	CAA.Jc	0.82		CAA.Qt.Vrt	0.62		CAA.Qt.Vrt	0.62
	VCA.MSsat	0.65		CAA.Qc	0.77		BSA.BsL	0.87		BSA.BsL	0.87
	VCA.ML.Hrz	1.00		CAA.Scpl	0.75		BSA.BCVL	1.00		BSA.BCVL	1.00
	VCA.sL.Hrz	0.67		CAA.Qcpl	0.68		BSA.BsSL	0.82		BSA.BsSL	0.82
	VCA.sL.Vrt	0.67		CAA.Jc.Hrz	0.82		BSA.BCVSL	0.99		BSA.BCVSL	0.99
	VCA.MDmax.Hrz	0.71		CAA.Jc.Vrt	0.82		BSA.BsL.Hrz	0.87		BSA.BsL.Hrz	0.87
	VCA.MDmax.Vrt	0.71		CAA.Qc.Hrz	0.97		BSA.BsL.Vrt	0.87		BSA.BsL.Vrt	0.87
	VCA.MSsat.Hrz	0.65		CAA.Scpl.Hrz	0.75		BSA.BCVL.Vrt	0.99		BSA.BCVL.Vrt	0.99
	VCA.MSsat.Vrt	0.65		CAA.Scpl.Vrt	0.76		BSA.BsSL.Hrz	0.82		BSA.BsSL.Hrz	0.82
				CAA.Qcpl.Hrz	0.69		BSA.BCVSL.Hrz	1.00		BSA.BCVSL.Hrz	1.00
				CAA.Qcpl.Vrt	0.71		BSA.BsSL.Vrt	0.82		BSA.BsSL.Vrt	0.82
			CAA.Qt	CAA.Qcpl	0.86		BSA.BCVSL.Vrt	0.98		BSA.BCVSL.Vrt	0.98
				CAA.Qt.Hrz	0.85	Col.kurtosis	Col.CoV	0.67	BSA.BCVSsat.Hrz	BSA.BCVDmax	0.80
				CAA.Qt.Vrt	0.84		Col.skew	0.93		BSA.BCVSsat	1.00
				CAA.Qcpl.Hrz	0.84		Col.CoV.hrz	0.70		BSA.BCVDmax.Hrz	0.80
				CAA.Qcpl.Vrt	0.83		Col.skew.hrz	0.94		BSA.BCVDmax.Vrt	0.79

	VCA.MSL.Vrt	VCA.MSL	1.00		Col.kurtosis.hrz	0.99		BSA.BCVSsat.Vrt	1.00
		VCA.sSL	0.83		Col.CoV.vrt	0.69	Lum.mean.vrt	Lum.mean	0.99
		VCA.MSL.Hrz	1.00		Col.skew.vrt	0.93		Lum.sd	0.94
		VCA.sSL.Hrz	0.83		Col.kurtosis.vrt	0.99		Lum.mean.hrz	0.96
		VCA.sSL.Vrt	0.83		BSA.BCVS	0.67		Lum.sd.hrz	0.90
					BSA.BCVS.Hrz	0.67		Lum.sd.vrt	0.97
					BSA.BCVS.Vrt	0.66		CAA.C	0.68
				Lum.CoV	Lum.CoV.hrz	0.93		CAA.C.Hrz	0.67
					Lum.CoV.vrt	0.93		CAA.C.Vrt	0.69
				Lum.kurtosis.vrt	Lum.skew	0.84	VCA.ML.Vrt	VCA.ML	1.00
					Lum.kurtosis	0.98		VCA.sL	0.67
					Lum.skew.hrz	0.71		VCA.MDmax	0.71
					Lum.kurtosis.hrz	0.85		VCA.MSsat	0.65
					Lum.skew.vrt	0.92		VCA.ML.Hrz	1.00
				VCA.CVS.Hrz	VCA.sS	0.62		VCA.sL.Hrz	0.67
					VCA.CVS	1.00		VCA.sL.Vrt	0.67
					VCA.sS.Hrz	0.62		VCA.MDmax.Hrz	0.71
					VCA.sS.Vrt	0.62		VCA.MDmax.Vrt	0.71
					VCA.CVS.Vrt	1.00		VCA.MSsat.Hrz	0.65
					BSA.BCVS	0.73		VCA.MSsat.Vrt	0.65
					BSA.BCVS.Hrz	0.73			
					BSA.BCVS.Vrt	0.73			
				VCA.MSL.Vrt	VCA.MSL	1.00			
					VCA.sSL	0.83			
					VCA.MSL.Hrz	1.00			
					VCA.sSL.Hrz	0.83			
					VCA.sSL.Vrt	0.83			

Figure S2

Schematic of the methodology.

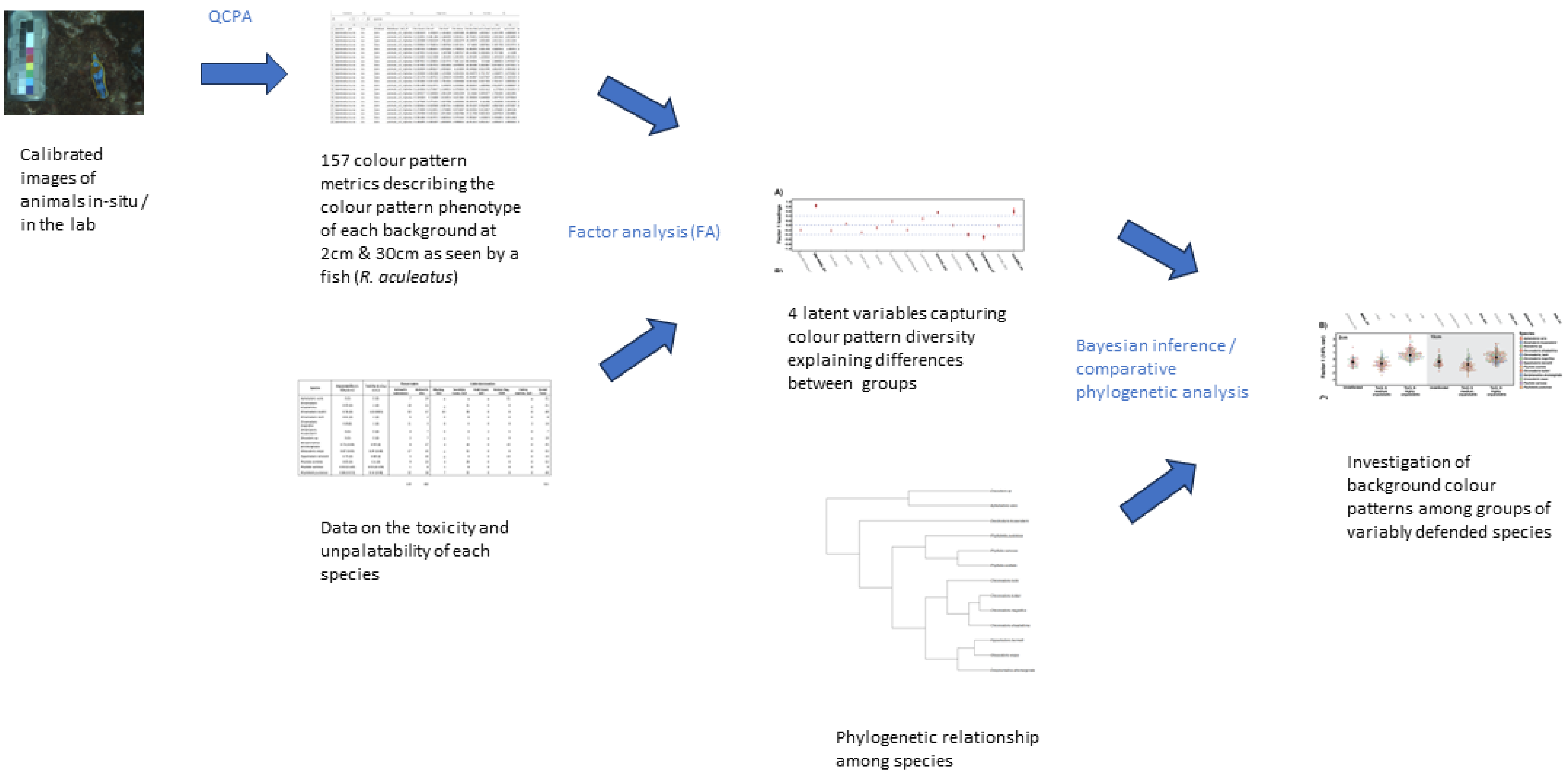

Figure S3

Parameters indicated with bold red writing in Fig. 3 are represented visually with their respective minimum and maximum value background in the dataset (A - D). (A) RNL chromaticity value (ΔS = Eurclidian distance from the achromatic point) image of the minimum (left) and maximum (right) Boundary Strength Analysis (BSA) coefficient of variation of the RNL saturation contrast between colour pattern elements in each background considered for horizontal transitions (BSA.BCVSsat.Hrz); (B) Zone map of the clustered backgrounds (k = cluster ID) showing the minimum (left) and maximum (right) relative Shannon colour diversity considering vertical transitions (CAA.Qc.vrt); (C) Local Edge Intensity image in RNL chromaticity contrast of the minimum (left) and maximum (right) kurtosis of the edge distribution (Col.kurtorsis). Different colours indicate edge orientations where brighter equals higher cumulative RNL chromaticity contrast (ΔS = Euclidian distance between pixels); (D) Original RAW image highlighting the minimum (left) and maximum (right) Visual Contrast Analysis abundance weighted mean luminance contrast (measured as the % double cone stimulation) considering vertical transitions (VCA.ML.vrt). The background considered for analysis is indicated with a red outline.

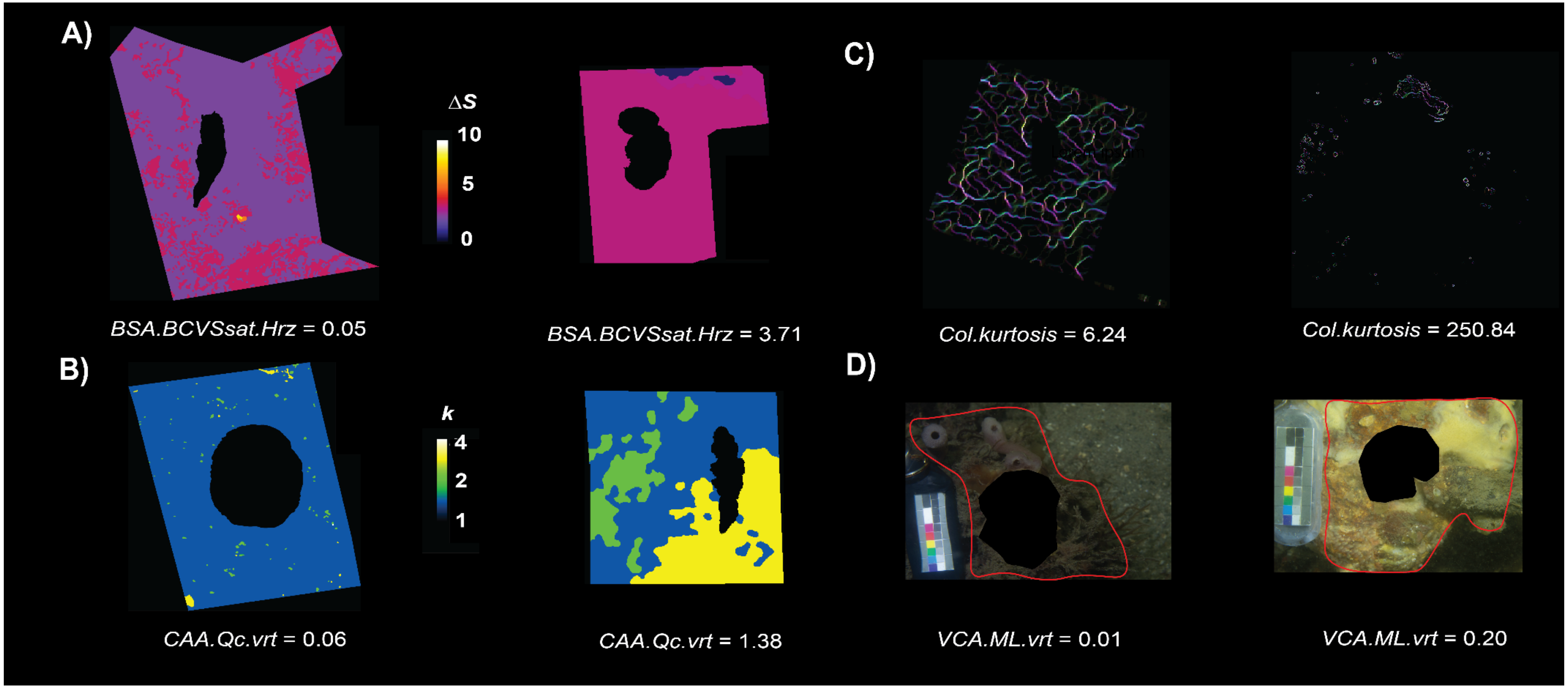

Figure S4

Respective minimum and maximum value background in the dataset for each factor. (A) Image of the minimum (left) and maximum (right) representative of Factor 1 shown as a RNL saturation image (ΔS = Euclidian distance from the achromatic point); (B) Zone map of the clustered backgrounds (k = cluster ID) showing the minimum (left) and maximum (right) representative of Factor 2; (C) Local Edge Intensity image in RNL chromaticity contrast of the minimum (left) and maximum (right) representative background for Factor 3. Different colours indicate edge orientations where brighter equals higher cumulative RNL chromaticity contrast (ΔS = Euclidian distance between pixels). (D) Original RAW image highlighting the minimum (left) and maximum (right) representative backgrounds for Factor 4.

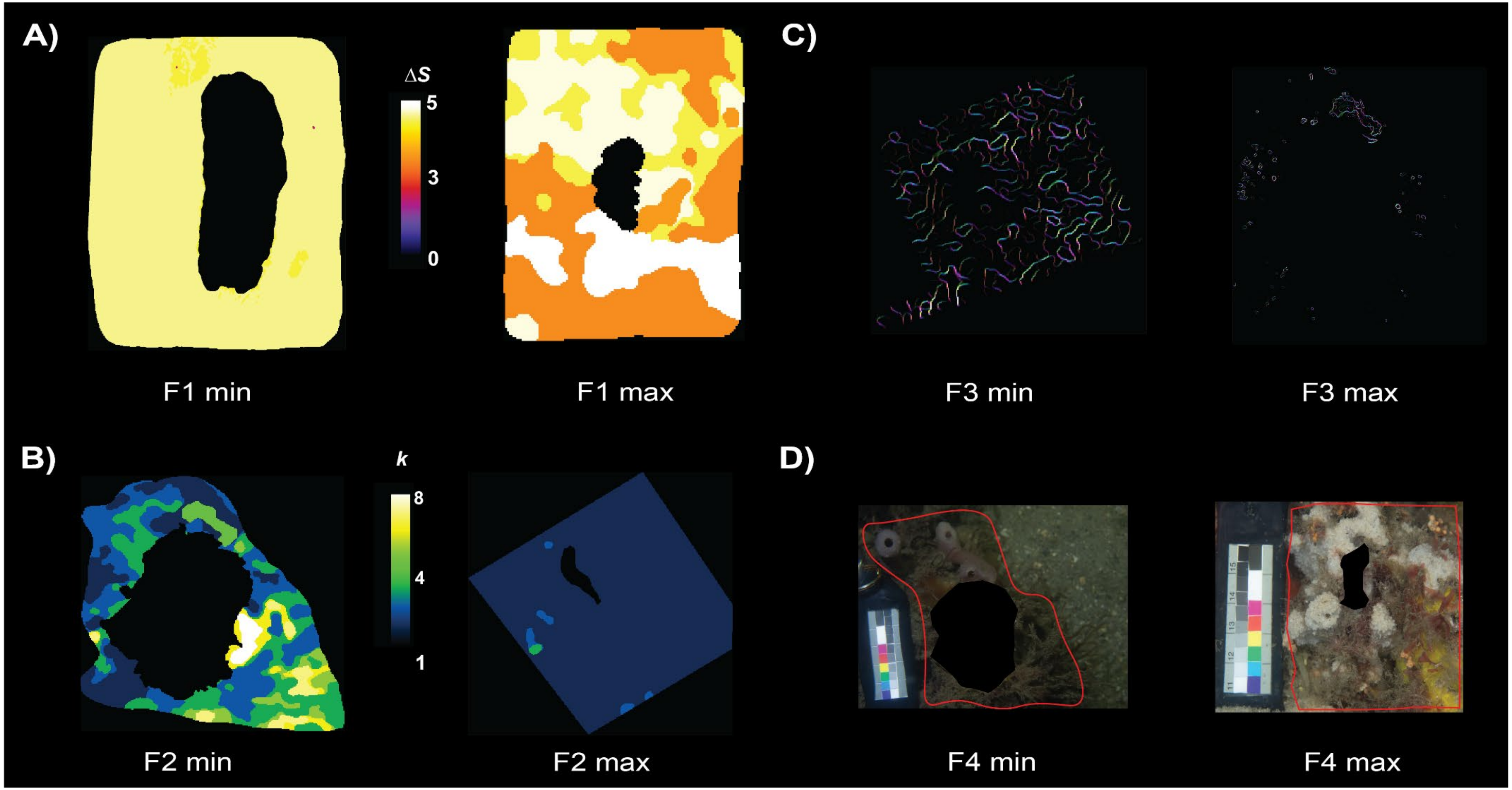

Table S4

Pairwise contrasts for the model investigating latent factor 1 expressed as the median differences between groups with different strengths of chemical defences (see 'Methods' for details). The effect size of pairwise differences increases with increasing deviation of such differences from zero, and the robustness of the result increases with decreasing degree of overlap of the 95% Credible Intervals (CIs) with zero.

**Factor 1: Contrasts**	**Difference**	**Lower CI**	**Upper CI**
Undefended at 2cm - Toxic and moderately unpalatable at 2cm	-1.53	-2.28	-0.78
Undefended at 2cm - Toxic and highly unpalatable at 2cm	-1.44	-2.18	-0.73
Undefended at 2cm - Undefended at 30cm	-0.18	-0.61	0.27
Undefended at 2cm - Toxic and moderately unpalatable at 30cm	-1.23	-1.99	-0.46
Undefended at 2cm - Toxic and highly unpalatable at 30cm	-1.35	-2.10	-0.65
Toxic and moderately unpalatable at 2cm - Toxic and highly unpalatable at 2cm	0.08	-0.38	0.54
Toxic and moderately unpalatable at 2cm - Undefended at 30cm	1.35	0.58	2.15
Toxic and moderately unpalatable at 2cm - Toxic and moderately unpalatable at 30cm	0.30	-0.02	0.63
Toxic and moderately unpalatable at 2cm - Toxic and highly unpalatable at 30cm	0.17	-0.28	0.65
Toxic and highly unpalatable at 2cm - Undefended at 30cm	1.26	0.47	2.04
Toxic and highly unpalatable at 2cm - Toxic and moderately unpalatable at 30cm	0.22	-0.29	0.70
Toxic and highly unpalatable at 2cm - Toxic and highly unpalatable at 30cm	0.09	-0.19	0.38
Undefended at 30cm - Toxic and moderately unpalatable at 30cm	-1.05	-1.83	-0.23
Undefended at 30cm - Toxic and highly unpalatable at 30cm	-1.17	-1.92	-0.37
Toxic and moderately unpalatable at 30cm - Toxic and highly unpalatable at 30cm	-0.13	-0.63	0.36

Table S5

Pairwise variance contrasts for the model investigating latent factor 1 expressed as the median differences of the residual standard deviation on the original scale (back-transformed from the log scale) between groups with different strengths of chemical defences (see 'Methods' for details). The effect size of pairwise differences increases with increasing deviation of such differences from zero, and the robustness of the result increases with decreasing degree of overlap of the 95% Credible Intervals (CIs) with zero.

**Factor 1: variance contrasts**	**Diference**	**Lower CI**	**Upper CI**
Undefended at 2cm - Toxic and moderately unpalatable at 2cm	0.21	-0.07	0.55
Undefended at 2cm - Toxic and highly unpalatable at 2cm	-0.03	-0.30	0.31
Undefended at 2cm - Undefended at 30cm	-0.12	-0.53	0.22
Undefended at 2cm - Toxic and moderately unpalatable at 30cm	-0.21	-0.67	0.12
Undefended at 2cm - Toxic and highly unpalatable at 30cm	0.13	-0.09	0.33
Toxic and moderately unpalatable at 2cm - Toxic and highly unpalatable at 2cm	-0.23	-0.49	0.01
Toxic and moderately unpalatable at 2cm - Undefended at 30cm	-0.33	-0.76	0.01
Toxic and moderately unpalatable at 2cm - Toxic and moderately unpalatable at 30cm	-0.42	-1.09	0.08
Toxic and moderately unpalatable at 2cm - Toxic and highly unpalatable at 30cm	-0.08	-0.45	0.22
Toxic and highly unpalatable at 2cm - Undefended at 30cm	-0.09	-0.52	0.25
Toxic and highly unpalatable at 2cm - Toxic and moderately unpalatable at 30cm	-0.19	-0.81	0.26
Toxic and highly unpalatable at 2cm - Toxic and highly unpalatable at 30cm	0.16	-0.26	0.51
Undefended at 30cm - Toxic and moderately unpalatable at 30cm	-0.09	-0.71	0.44
Undefended at 30cm - Toxic and highly unpalatable at 30cm	0.25	-0.15	0.69
Toxic and moderately unpalatable at 30cm - Toxic and highly unpalatable at 30cm	0.34	-0.06	0.85

Table S6

Pairwise contrasts for the model investigating latent factor 2 expressed as the median differences between groups with different strengths of chemical defences (see ‘Methods’ for details). The effect size of pairwise differences increases with increasing deviation of such differences from zero, and the robustness of the result increases with decreasing degree of overlap of the 95% Credible Intervals (CIs) with zero.

Factor 2: Contrasts	Difference	Lower CI	Upper CI
Undefended at 2cm - Toxic and moderately unpalatable at 2cm	-0.1226	-0.614	0.402
Undefended at 2cm - Toxic and highly unpalatable at 2cm	-0.1056	-0.617	0.394
Undefended at 2cm - Undefended at 30cm	1.0072	0.568	1.482
Undefended at 2cm - Toxic and moderately unpalatable at 30cm	0.8871	0.352	1.417
Undefended at 2cm - Toxic and highly unpalatable at 30cm	0.6930	0.125	1.194
Toxic and moderately unpalatable at 2cm - Toxic and highly unpalatable at 2cm	0.0175	-0.333	0.347
Toxic and moderately unpalatable at 2cm - Undefended at 30cm	1.1337	0.552	1.705
Toxic and moderately unpalatable at 2cm - Toxic and moderately unpalatable at 30cm	1.0052	0.668	1.312
Toxic and moderately unpalatable at 2cm - Toxic and highly unpalatable at 30cm	0.8158	0.410	1.164
Toxic and highly unpalatable at 2cm - Undefended at 30cm	1.1151	0.552	1.686
Toxic and highly unpalatable at 2cm - Toxic and moderately unpalatable at 30cm	0.9896	0.615	1.363
Toxic and highly unpalatable at 2cm - Toxic and highly unpalatable at 30cm	0.7997	0.473	1.105
Undefended at 30cm - Toxic and moderately unpalatable at 30cm	-0.1239	-0.709	0.462
Undefended at 30cm - Toxic and highly unpalatable at 30cm	-0.3172	-0.971	0.230
Toxic and moderately unpalatable at 30cm - Toxic and highly unpalatable at 30cm	-0.1898	-0.615	0.215

Table S7

Pairwise variance contrasts for the model investigating latent factor 2 expressed as the median differences of the residual standard deviation on the original scale (back-transformed from the log scale) between groups with different strengths of chemical defences (see 'Methods' for details). The effect size of pairwise differences increases with increasing deviation of such differences from zero, and the robustness of the result increases with decreasing degree of overlap of the 95% Credible Intervals (CIs) with zero.

Factor 2: Variance Contrast	Difference	Lower CI	Upper CI
Undefended at 2cm - Toxic and moderately unpalatable at 2cm	0.16	-0.08	0.45
Undefended at 2cm - Toxic and highly unpalatable at 2cm	0.06	-0.19	0.33
Undefended at 2cm - Undefended at 30cm	-0.12	-0.48	0.21
Undefended at 2cm - Toxic and moderately unpalatable at 30cm	0.01	-0.31	0.26
Undefended at 2cm - Toxic and highly unpalatable at 30cm	-0.13	-0.43	0.11
Toxic and moderately unpalatable at 2cm - Toxic and highly unpalatable at 2cm	-0.11	-0.32	0.11
Toxic and moderately unpalatable at 2cm - Undefended at 30cm	-0.28	-0.67	0.04
Toxic and moderately unpalatable at 2cm - Toxic and moderately unpalatable at 30cm	-0.15	-0.65	0.24
Toxic and moderately unpalatable at 2cm - Toxic and highly unpalatable at 30cm	-0.29	-0.73	0.05
Toxic and highly unpalatable at 2cm - Undefended at 30cm	-0.18	-0.55	0.14
Toxic and highly unpalatable at 2cm - Toxic and moderately unpalatable at 30cm	-0.05	-0.47	0.28
Toxic and highly unpalatable at 2cm - Toxic and highly unpalatable at 30cm	-0.18	-0.67	0.21
Undefended at 30cm - Toxic and moderately unpalatable at 30cm	0.13	-0.34	0.55
Undefended at 30cm - Toxic and highly unpalatable at 30cm	0.00	-0.47	0.43
Toxic and moderately unpalatable at 30cm - Toxic and highly unpalatable at 30cm	-0.13	-0.53	0.27

Table S8

Pairwise contrasts for the model investigating latent factor 3 expressed as the median differences between groups with different strengths of chemical defences (see 'Methods' for details). The effect size of pairwise differences increases with increasing deviation of such differences from zero, and the robustness of the result increases with decreasing degree of overlap of the 95% Credible Intervals (CIs) with zero.

**Factor 3: Contrasts**	**Difference**	**Lower CI**	**Upper CI**
Undefended at 2cm - Toxic and moderately unpalatable at 2cm	0.23	-0.27	0.69
Undefended at 2cm - Toxic and highly unpalatable at 2cm	0.18	-0.26	0.66
Undefended at 2cm - Undefended at 30cm	0.50	0.07	0.91
Undefended at 2cm - Toxic and moderately unpalatable at 30cm	0.50	0.00	1.01
Undefended at 2cm - Toxic and highly unpalatable at 30cm	0.51	0.02	0.97
Toxic and moderately unpalatable at 2cm - Toxic and highly unpalatable at 2cm	-0.05	-0.37	0.24
Toxic and moderately unpalatable at 2cm - Undefended at 30cm	0.26	-0.31	0.82
Toxic and moderately unpalatable at 2cm - Toxic and moderately unpalatable at 30cm	0.27	-0.03	0.56
Toxic and moderately unpalatable at 2cm - Toxic and highly unpalatable at 30cm	0.28	-0.08	0.60
Toxic and highly unpalatable at 2cm - Undefended at 30cm	0.32	-0.24	0.84
Toxic and highly unpalatable at 2cm - Toxic and moderately unpalatable at 30cm	0.32	-0.02	0.69
Toxic and highly unpalatable at 2cm - Toxic and highly unpalatable at 30cm	0.33	0.07	0.56
Undefended at 30cm - Toxic and moderately unpalatable at 30cm	0.01	-0.59	0.60
Undefended at 30cm - Toxic and highly unpalatable at 30cm	0.01	-0.53	0.60
Toxic and moderately unpalatable at 30cm - Toxic and highly unpalatable at 30cm	0.01	-0.35	0.40

Table S9

Pairwise variance contrasts for the model investigating latent factor 3 expressed as the median differences of the residual standard deviation on the original scale (back-transformed from the log scale) between groups with different strengths of chemical defences (see 'Methods' for details). The effect size of pairwise differences increases with increasing deviation of such differences from zero, and the robustness of the result increases with decreasing degree of overlap of the 95% Credible Intervals (CIs) with zero.

Factor 3: Variance Contrast	Estimate	Lower CI	Upper CI
Undefended at 2cm - Toxic and moderately unpalatable at 2cm	0.15	-0.10	0.44
Undefended at 2cm - Toxic and highly unpalatable at 2cm	0.09	-0.15	0.38
Undefended at 2cm - Undefended at 30cm	-0.19	-0.62	0.15
Undefended at 2cm - Toxic and moderately unpalatable at 30cm	-0.12	-0.46	0.14
Undefended at 2cm - Toxic and highly unpalatable at 30cm	0.00	-0.24	0.19
Toxic and moderately unpalatable at 2cm - Toxic and highly unpalatable at 2cm	-0.06	-0.26	0.14
Toxic and moderately unpalatable at 2cm - Undefended at 30cm	-0.34	-0.78	-0.01
Toxic and moderately unpalatable at 2cm - Toxic and moderately unpalatable at 30cm	-0.27	-0.78	0.15
Toxic and moderately unpalatable at 2cm - Toxic and highly unpalatable at 30cm	-0.15	-0.55	0.15
Toxic and highly unpalatable at 2cm - Undefended at 30cm	-0.28	-0.72	0.05
Toxic and highly unpalatable at 2cm - Toxic and moderately unpalatable at 30cm	-0.21	-0.67	0.15
Toxic and highly unpalatable at 2cm - Toxic and highly unpalatable at 30cm	-0.09	-0.53	0.25
Undefended at 30cm - Toxic and moderately unpalatable at 30cm	0.08	-0.43	0.58
Undefended at 30cm - Toxic and highly unpalatable at 30cm	0.19	-0.23	0.66
Toxic and moderately unpalatable at 30cm - Toxic and highly unpalatable at 30cm	0.11	-0.23	0.51

Table S10

Pairwise contrasts for the model investigating latent factor 4 expressed as the median differences between groups with different strengths of chemical defences (see ‘Methods’ for details). The effect size of pairwise differences increases with increasing deviation of such differences from zero, and the robustness of the result increases with decreasing degree of overlap of the 95% Credible Intervals (CIs) with zero.

Factor 4: Contrasts	Estimate	Lower CI	Upper CI
Undefended at 2cm - Toxic and moderately unpalatable at 2cm	0.21	-0.51	0.96
Undefended at 2cm - Toxic and highly unpalatable at 2cm	0.31	-0.38	1.04
Undefended at 2cm - Undefended at 30cm	0.21	-0.25	0.69
Undefended at 2cm - Toxic and moderately unpalatable at 30cm	0.75	0.02	1.48
Undefended at 2cm - Toxic and highly unpalatable at 30cm	0.73	0.05	1.51
Toxic and moderately unpalatable at 2cm - Toxic and highly unpalatable at 2cm	0.09	-0.31	0.53
Toxic and moderately unpalatable at 2cm - Undefended at 30cm	-0.01	-0.81	0.78
Toxic and moderately unpalatable at 2cm - Toxic and moderately unpalatable at 30cm	0.53	0.25	0.81
Toxic and moderately unpalatable at 2cm - Toxic and highly unpalatable at 30cm	0.52	0.08	0.98
Toxic and highly unpalatable at 2cm - Undefended at 30cm	-0.10	-0.92	0.65
Toxic and highly unpalatable at 2cm - Toxic and moderately unpalatable at 30cm	0.44	0.02	0.84
Toxic and highly unpalatable at 2cm - Toxic and highly unpalatable at 30cm	0.42	0.16	0.70
Undefended at 30cm - Toxic and moderately unpalatable at 30cm	0.54	-0.26	1.35
Undefended at 30cm - Toxic and highly unpalatable at 30cm	0.53	-0.27	1.32
Toxic and moderately unpalatable at 30cm - Toxic and highly unpalatable at 30cm	-0.02	-0.42	0.44

Table S11

Pairwise variance contrasts for the model investigating latent factor 4 expressed as the median differences of the residual standard deviation on the original scale (back-transformed from the log scale) between groups with different strengths of chemical defences (see 'Methods' for details). The effect size of pairwise differences increases with increasing deviation of such differences from zero, and the robustness of the result increases with decreasing degree of overlap of the 95% Credible Intervals (CIs) with zero.

Factor 4: Variance Contrasts	Estimate	Lower CI	Upper CI
Undefended at 2cm - Toxic and moderately unpalatable at 2cm	0.08	-0.19	0.40
Undefended at 2cm - Toxic and highly unpalatable at 2cm	0.07	-0.19	0.39
Undefended at 2cm - Undefended at 30cm	-0.24	-0.66	0.11
Undefended at 2cm - Toxic and moderately unpalatable at 30cm	0.21	0.00	0.40
Undefended at 2cm - Toxic and highly unpalatable at 30cm	-0.05	-0.31	0.14
Toxic and moderately unpalatable at 2cm - Toxic and highly unpalatable at 2cm	-0.01	-0.24	0.23
Toxic and moderately unpalatable at 2cm - Undefended at 30cm	-0.32	-0.80	0.04
Toxic and moderately unpalatable at 2cm - Toxic and moderately unpalatable at 30cm	0.13	-0.27	0.46
Toxic and moderately unpalatable at 2cm - Toxic and highly unpalatable at 30cm	-0.13	-0.56	0.21
Toxic and highly unpalatable at 2cm - Undefended at 30cm	-0.31	-0.78	0.04
Toxic and highly unpalatable at 2cm - Toxic and moderately unpalatable at 30cm	0.14	-0.20	0.40
Toxic and highly unpalatable at 2cm - Toxic and highly unpalatable at 30cm	-0.12	-0.60	0.25
Undefended at 30cm - Toxic and moderately unpalatable at 30cm	0.45	0.07	0.90
Undefended at 30cm - Toxic and highly unpalatable at 30cm	0.19	-0.25	0.65
Toxic and moderately unpalatable at 30cm - Toxic and highly unpalatable at 30cm	-0.26	-0.59	0.03

Table S12

Coefficient estimates of the model investigating the scores for latent factor 1 between species of nudibranchs with different levels of chemical defences (N = 12, R^2 = 0.36). Estimates are based on a Student distribution with an identity link for the mean of the response distribution and a log link for its residual standard deviation (Sigma). The estimate is more likely to be non-zero when the credible intervals do not overlap with zero.

Coefficient	Mean	M. Error	95% CIs	
			Low	High
**Group-level effects**				
Phylogenesis				
Sd [Intercept]	0.24	0.18	0.01	0.66
Species of nudibranch				
Sd [Intercept: Distance 2 cm]	0.21	0.12	0.02	0.48
Sd [Distance 30 cm]	0.12	0.10	0.00	0.36
Sigma: Sd [Intercept: Distance 2 cm]	0.12	0.09	0.00	0.35
Sigma: Sd [Distance 30 cm]	0.14	0.11	0.01	0.41
**Population-level effects**				
Intercept [Undefended, 2 cm]	-1.08	0.28	-1.63	-0.51
Sigma_Intercept [Undefended, 2 cm]	-0.24	0.16	-0.53	0.08
Chemical defence [Toxic and moderately unpalatable]	1.52	0.37	0.73	2.24
Chemical defence [Toxic and highly unpalatable]	1.44	0.36	0.73	2.18
Distance [30 cm]	0.18	0.22	-0.25	0.63
Chemical defence [Toxic and moderately unpalatable] x Distance [30 cm]	-0.48	0.28	-1.02	0.07
Chemical defence [Toxic and highly unpalatable] x Distance [30 cm]	-0.27	0.27	-0.80	0.25
Sigma: Chemical defence [Toxic and moderately unpalatable]	-0.30	0.21	-0.76	0.1
Sigma: Chemical defence [Toxic and highly unpalatable]	0.04	0.19	-0.36	0.38
Sigma: Distance [30cm]	0.13	0.21	-0.27	0.55
Sigma: Chemical defence [Toxic and moderately unpalatable] x Distance [30 cm]	0.09	0.28	-0.47	0.65
Sigma: Chemical defence [Toxic and highly unpalatable] x Distance [30 cm]	-0.32	0.26	-0.85	0.18

Table S13

Coefficient estimates of the model investigating the scores for latent factor 2 between species of nudibranchs with different levels of chemical defences (N = 12, R^2 = 0.28). Estimates are based on a Student distribution with an identity link for the mean of the response distribution and a log link for its residual standard deviation (Sigma). The estimate is more likely to be non-zero when the credible intervals do not overlap with zero.

**Coefficient**	**Mean**	**M. Error**	**95% CIs**	
			**Low**	**High**
**Group-level effects**				
Phylogenesis				
Sd [Intercept]	0.13	0.11	0	0.42
Species of nudibranch				
Sd [Intercept: Distance 2 cm]	0.14	0.09	0.01	0.35
Sd [Distance 30 cm]	0.16	0.12	0.01	0.44
Sigma: Sd [Intercept: Distance 2 cm]	0.09	0.08	0.00	0.28
Sigma: Sd [Distance 30 cm]	0.12	0.10	0.00	0.38
**Population-level effects**				
Intercept [Undefended, 2 cm]	0.31	0.20	-0.09	0.7
Sigma_Intercept [Undefended, 2 cm]	-0.29	0.14	-0.57	0
Chemical defence [Toxic and moderately unpalatable]	0.12	0.26	-0.39	0.63
Chemical defence [Toxic and highly unpalatable]	0.11	0.26	-0.38	0.63
Distance [30 cm]	-1.01	0.23	-1.48	-0.6
Chemical defence [Toxic and moderately unpalatable] x Distance [30 cm]	0.00	0.28	-0.56	0.56
Chemical defence [Toxic and highly unpalatable] x Distance [30 cm]	0.21	0.28	-0.33	0.78
Sigma: Chemical defence [Toxic and moderately unpalatable]	-0.24	0.19	-0.63	0.13
Sigma: Chemical defence [Toxic and highly unpalatable]	-0.07	0.17	-0.42	0.26
Sigma: Distance [30cm]	0.14	0.21	-0.28	0.54
Sigma: Chemical defence [Toxic and moderately unpalatable] x Distance [30 cm]	-0.17	0.27	-0.70	0.38
Sigma: Chemical defence [Toxic and highly unpalatable] x Distance [30 cm]	0.00	0.25	-0.48	0.5

Table S14

Coefficient estimates of the model investigating the scores for latent factor 3 between species of nudibranchs with different levels of chemical defences (N = 12, R^2 = 0.07). Estimates are based on a Student distribution with an identity link for the mean of the response distribution and a log link for its residual standard deviation (Sigma). The estimate is more likely to be non-zero when the credible intervals do not overlap with zero.

**Coefficient**	**Mean**	**M. Error**	**95% CIs**	
			**Low**	**High**
**Group-level effects**				
Phylogenesis				
Sd [Intercept]	0.11	0.11	0	0.39
Species of nudibranch				
Sd [Intercept: Distance 2 cm]	0.13	0.08	0.01	0.32
Sd [Distance 30 cm]	0.12	0.10	0.00	0.36
Sigma: Sd [Intercept: Distance 2 cm]	0.13	0.09	0.01	0.35
Sigma: Sd [Distance 30 cm]	0.12	0.10	0.00	0.36
**Population-level effects**				
Intercept [Undefended, 2 cm]	0.22	0.18	-0.14	0.58
Sigma_Intercept [Undefended, 2 cm]	-0.49	0.19	-0.87	-0.1
Chemical defence [Toxic and moderately unpalatable]	-0.23	0.24	-0.69	0.27
Chemical defence [Toxic and highly unpalatable]	-0.18	0.23	-0.63	0.3
Distance [30 cm]	-0.49	0.22	-0.91	-0.07
Chemical defence [Toxic and moderately unpalatable] x Distance [30 cm]	0.23	0.27	-0.30	0.75
Chemical defence [Toxic and highly unpalatable] x Distance [30 cm]	0.17	0.25	-0.32	0.66
Sigma: Chemical defence [Toxic and moderately unpalatable]	-0.27	0.24	-0.74	0.19
Sigma: Chemical defence [Toxic and highly unpalatable]	-0.14	0.22	-0.60	0.29
Sigma: Distance [30cm]	0.26	0.25	-0.22	0.75
Sigma: Chemical defence [Toxic and moderately unpalatable] x Distance [30 cm]	-0.11	0.32	-0.75	0.51
Sigma: Chemical defence [Toxic and highly unpalatable] x Distance [30 cm]	-0.27	0.30	-0.87	0.32

Table S15

Coefficient estimates of the model investigating the scores for latent factor 4 between species of nudibranchs with different levels of chemical defences (N = 12, $R^2 = 0.15$). Estimates are based on a Student distribution with an identity link for the mean of the response distribution and a log link for its residual standard deviation (Sigma). The estimate is more likely to be non-zero when the credible intervals do not overlap with zero.

Coefficient	Mean	M. Error	95% CIs	
			Low	High
**Group-level effects**				
Phylogenesis				
Sd [Intercept]	0.23	0.18	0.01	0.66
Species of nudibranch				
Sd [Intercept: Distance 2 cm]	0.19	0.11	0.02	0.44
Sd [Distance 30 cm]	0.11	0.09	0.00	0.33
Sigma: Sd [Intercept: Distance 2 cm]	0.12	0.09	0.01	0.33
Sigma: Sd [Distance 30 cm]	0.10	0.08	0.00	0.3
**Population-level effects**				
Intercept [Undefended, 2 cm]	0.43	0.27	-0.09	0.98
Sigma_Intercept [Undefended, 2 cm]	-0.31	0.17	-0.64	0.04
Chemical defence [Toxic and moderately unpalatable]	-0.22	0.36	-0.99	0.49
Chemical defence [Toxic and highly unpalatable]	-0.32	0.36	-1.07	0.36
Distance [30 cm]	-0.21	0.24	-0.67	0.27
Chemical defence [Toxic and moderately unpalatable] x Distance [30 cm]	-0.33	0.27	-0.87	0.21
Chemical defence [Toxic and highly unpalatable] x Distance [30 cm]	-0.22	0.27	-0.75	0.31
Sigma: Chemical defence [Toxic and moderately unpalatable]	-0.11	0.21	-0.54	0.29
Sigma: Chemical defence [Toxic and highly unpalatable]	-0.09	0.20	-0.50	0.29
Sigma: Distance [30cm]	0.28	0.21	-0.13	0.69
Sigma: Chemical defence [Toxic and moderately unpalatable] x Distance [30 cm]	-0.62	0.27	-1.15	-0.09
Sigma: Chemical defence [Toxic and highly unpalatable] x Distance [30 cm]	-0.22	0.25	-0.72	0.27
